# The prey of the Harpy Eagle in its last reproductive refuges in the Atlantic Forest

**DOI:** 10.1038/s41598-023-44014-9

**Published:** 2023-10-25

**Authors:** Mylena Kaizer, Brener Fabres, Francisca Helena Aguiar-Silva, Tânia Margarete Sanaiotti, Alexandro Ribeiro Dias, Aureo Banhos

**Affiliations:** 1 Projeto Harpia - Mata Atlântica (Harpy Eagle Project - Atlantic Forest), Universidade Federal do Espírito Santo - UFES, Alto Universitário, Guararema, Alegre, Espírito Santo 29500-000 Brazil; 2 Projeto Harpia (Harpy Eagle Project - Brazil), Instituto Nacional de Pesquisas da Amazônia - INPA, Av. André Araújo, 2936, Aleixo, Manaus, Amazonas 69067-375 Brazil; 3 Programa de Pós-Graduação em Zoologia - PPGZOOL, Universidade Federal do Amazonas - UFAM, Av. General Rodrigo Otávio Jordão Ramos, 3000, Coroado, Manaus, Amazonas 69077-000 Brazil; 4 Programa de Pós-Graduação em Ciências Biológicas (Biologia Animal) - PPGBAN, Universidade Federal do Espírito Santo - UFES, Avenida Fernando Ferrari, 514, Prédio Barbara Weinberg, Vitória, Espírito Santo 29075-910 Brazil; 5 Coordenação de Biodiversidade, Instituto Nacional de Pesquisas da Amazônia - INPA, Avenida André Araújo, 2936, Petrópolis, Manaus, Amazonas 69067-375 Brazil; 6 Reserva Particular de Patrimônio Natural Estação Veracel, Rodovia BR-367, 37, Porto Seguro, Bahia 45810-000 Brazil; 7 Departamento de Biologia, Centro de Ciências Exatas, Naturais e da Saúde, Universidade Federal do Espírito Santo - UFES, Alto Universitário, Guararema, Alegre, Espírito Santo 29500-000 Brazil

**Keywords:** Behavioural ecology, Biodiversity, Forest ecology

## Abstract

The Harpy Eagle (*Harpia harpyja*) is threatened with extinction throughout its distribution in the neotropical forests. In the Atlantic Forest, deforestation has reduced the number of suitable habitats, with only a few remnant forest fragments hosting active nests; currently, the only known nests in this region are in the Central Atlantic Forest Ecological Corridor (CAFEC), in Brazil. Little is known about Harpy Eagle diets in this region, despite this information being essential for developing effective conservation strategies. We classified the composition, frequency, richness, ecological attributes, and conservation status of the species that make up the Harpy Eagle’s diet in its last refuges in the CAFEC. Between 2017 and 2021, we collected and analyzed 152 prey remains and 285 camera trap photographs from seven active nests. We identified at least 16 mammal species (96.7%), one parrot and other bird remains (3.3%). The Harpy Eagle’s diet consisted mainly of medium-sized arboreal, folivorous, frugivorous, and diurnal mammals. Five prey species are currently threatened with extinction at global, six at national and seven at regional levels. The majority of the diet consists of *Sapajus robustus*, which is threatened, and *Bradypus variegatus*, which is not threatened. In addition to the effects of habitat loss and hunting, the Harpy Eagle may also suffer from the decline in the populations of their prey in the Atlantic Forest.

## Introduction

**Figure 1:**
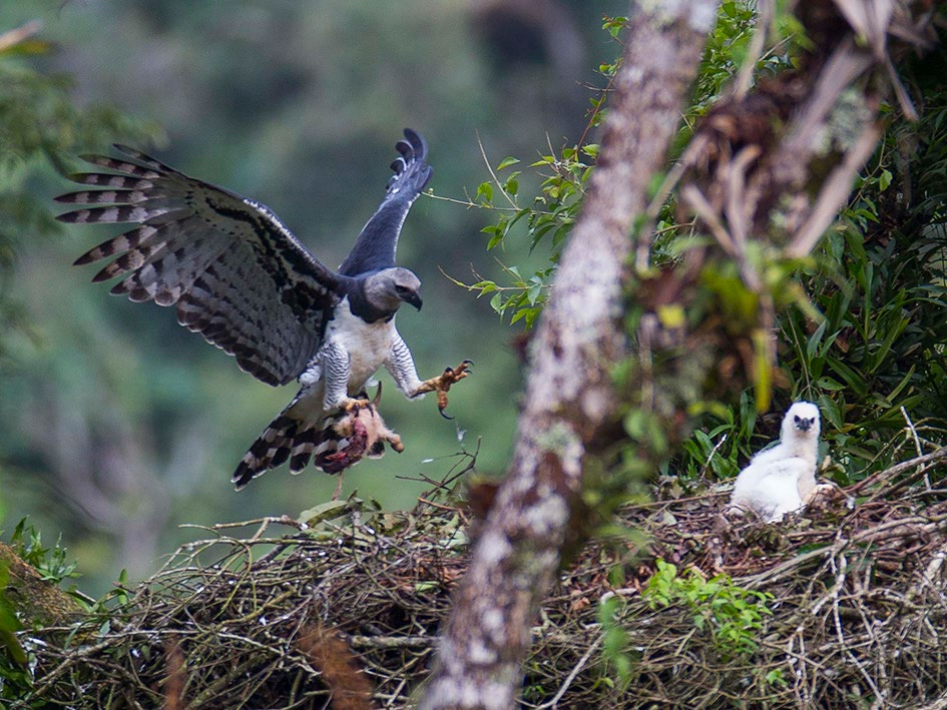
Adult Harpy Eagle arriving at the nest with an southern tamandua (*Tamandua tetradactyla*) prey to feed the eaglet. The nest was monitored in the Serra Bonia Private Natural Heritage Reserve, in Bahia, for this study. Photo by João Marcos Rosa.

The Harpy Eagle (*Harpia harpyja*) (Fig. 1) is one of the largest eagles in the world. It is found in the neotropical forests of Central and South America, mainly in the Brazilian Amazon and Atlantic forests, and depends on forests for reproduction and foraging^1,2^. Breeding pairs nest in emergent and canopy trees standing over 40 m in height and usually they returns to the same tree to nest throughout their reproductive life^3–6^. The Harpy Eagle forages across a large area around its nest^7,8^, and feeds mainly on arboreal prey, such as sloths and monkeys^9–11^.

The Harpy Eagle is threatened with extinction, specifically in the Vulnerable category^2^. In Brazil, it is also classified as Vulnerable^12^. Habitat loss, hunting, and persecution comprise the major threats to wild Harpy Eagle populations^2,13,14^. They are sensitive to habitat modifications, and the problem is confounded by their low population density and reproductive rates, producing only one eaglet every 2.5–3 years^4,9,15^.

In the Brazilian Atlantic Forest, the Harpy Eagle’ situation is more alarming^16^, it is classified as Critically Endangered in most states that constitute its range including Espírito Santo (ES)^17^ and Bahia (BA)^18^. The Atlantic Forest is a global biodiversity hotspot^19^, yet over 85% of the original forest has been lost^20^. Although records of individual Harpy Eagles are scattered throughout the region^21–28^, nesting records are rare, and located primarily in the Misiones region of Argentina^29^. The most recent nest records in protected areas are in the Central Atlantic Forest Ecological Corridor (CAFEC) in northern ES^30^ and southern BA^26,31^.

The loss of forest cover also impacts species populations that make up the diet of the Harpy Eagle, which means resource limitations for maintaining the population of this predator. This is because some of the prey species are also dependent on intact, hunting-free forests to survive. Therefore, if prey species disappear due to habitat degradation, it can lead to the disruption of food chains and dietary overlap among species that previously did not compete for the same food resources, causing an ecological imbalance^32^.

Studies on the Harpy Eagle’s diet have been conducted mainly in the Amazon^9–11,15,33,34^ and in the Atlantic Forest, some information on the Harpy Eagle’s diet has been gathered from studies conducted in Argentina^29^ and Brazil^22,30,31^. However, no study has focused on the Harpy Eagle’s feeding habits in this region, where both the predator and its potential prey are dramatically at risk of extinction. In this study, we investigated the Harpy Eagle’s feeding habits in protected areas in the CAFEC and assessed the composition, foraging stratum, diet, estimated body mass, and conservation status of its prey.

## Materials and methods

### Study area

**Figure 2:**
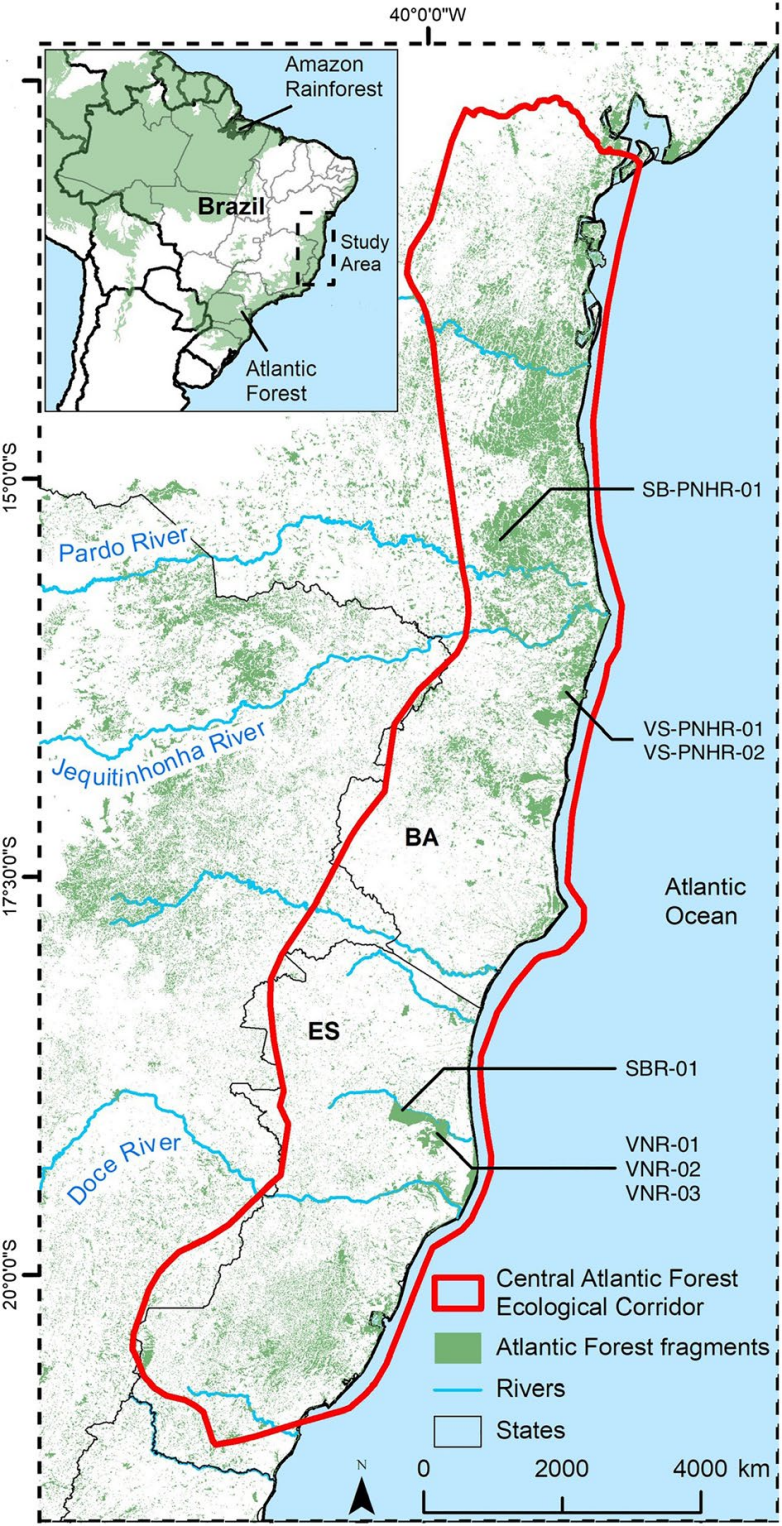
Location of Harpy Eagle nests monitored in Central Atlantic Forest Ecological Corridor reserves. Abbreviations: Bahia (BA), Espírito Santo (ES), Serra Bonita Private Natural Heritage Reserve (SB-PNHR), Sooretama Biological Reserve (SBR), Vale Natural Reserve (VNR), Veracel Station Private Natural Heritage Reserve (VS-PNHR). The map was made using with licensed software ArcGIS Pro version 10.8.2 (https://www.esri.com/en-us/arcgis/products/arcgis-pro/overview).

The study was conducted in four protected areas located in the CAFEC in southeastern and northeastern Brazil (Fig. 2): Vale Natural Reserve (VNR) and Sooretama Biological Reserve (SBR) are contiguous and have areas of 22,711 ha and 27,558 ha, respectively, and are located in the drainage basins of the Doce river in northern ES; Veracel Station Private Natural Heritage Reserve (VS-PNHR) covers 6069 ha in the basin of the Jequitinhonha river in southern BA; and Serra Bonita Private Natural Heritage Reserve (SB-PNHR) is a group of preserved rural properties which form an area of 2500 ha in the basin of the Pardo river in southern BA.

We monitored seven active Harpy Eagle nests in the CAFEC. Although the Harpy Eagle nests monitored in this study are in forested areas, it is important to highlight that each nest has its own surrounding vegetative landscape. In the VNR was monitored three nests of the species (VNR-01, VNR-02 and VNR-03). The VNR-01 nest is located approximately 600 m away from pasture areas and land intended for growing papaya and coffee. The VNR-02 nest is located approximately 1 km away from the crops, while the VNR-03 nest is further from the edge, it is approximately 5 km away from these cultivation areas. In the SBR, only one nest was monitored (SBR-01), which is located approximately 2.5 km away from BR-101 highway. Regarding VS-PNHR, two nests were monitored (VS-PNHR-01 and VS-PNHR-02), which are approximately 2 km away each from the pasture and eucalyptus plantation areas. Finally, in the SB-PNHR, only one nest was monitored (SB-PNHR-01), which is located approximately 400 m away to the cultivation areas, where cocoa was planted in the shade of the native forest, known as cabrucas.

### Data collection

We collected our data between March 2017 and February 2021 (Table 1). During the study, the seven pairs of Harpy Eagles were at different stages of the reproductive cycle. In VNR-01 nest, we recorded two unsuccessful breeding attempts and one successful. In the latter, we monitored the development of the eaglet from birth (February 2019) until the two years old. In VNR-02 nest, the couple frequented the nest and hatched an egg, but the eaglet was not born. In VNR-03 nest, although the couple visited the nest and began the courtship period, no eggs were laid during the monitoring period. In SBR-01 nest, we monitored a five-month-old eaglet until the end of the reproductive cycle (September 2017–January 2019), when it left the nest. In a second cycle, the couple from the SBR-01 nest tried to reproduce again, but the eaglet died less than month old (October 2019–January 2020). In VS-PNHR-01 nest, we monitored the final stage of the couple’s reproductive cycle, with the eaglet outside the nest but spotted nearby. In VS-PNHR-02 nest, we monitored a four-month-old eaglet until the end of the reproductive cycle (May 2018–September 2019). In SB-PNHR-01 nest, we monitored a female incubating the egg until the end of the reproductive cycle (July 2018–January 2020).

**Table 1:** Monitoring effort of the seven Harpy Eagle nests in the north of Espírito Santo (ES) and south of Bahia (BA), in the Central Atlantic Forest Ecological Corridor.

Region	Nest	Eaglet	Sampling period	Prey remains			Camera trap		
				Visits	Presence	Absence	Records	Effort	Success
ES	VNR-01	Presence	15/03/2017–27/02/2021	59	39	20	147	1085	13.55
ES	VNR-02	Absence	04/11/2017–27/02/2021	37	27	15	26	866	3.00
ES	VNR-03	Absence	20/07/2019–27/02/2021	16	4	33	2	410	0.49
ES	SBR-01	Presence	08/08/2017–28/02/2021	42	9	7	70	1099	6.37
BA	VS-PNHR-01	Absence	07/04/2018–25/02/2021	45	26	19	1	601	0.17
BA	VS-PNHR-02	Presence	23/05/2018–26/02/2021	43	34	9	24	488	4.92
BA	SB-PNHR-01	Presence	03/06/2018–23/02/2021	33	13	20	15	399	3.76

Nests with presence and absence of eaglet, sampling period, total number of visits and the number of visits with presence and absence of prey remains below each nest, and number of records, records effort (day camera) and records success of the camera trap (%).

We installed a nylon shade cloth under each nest, about 10–20 m^2^ and 1.30 m above the ground, to intercept prey remains that fell from nests, such as bone fragments, unconsumed prey remains, and regurgitated pellets^33^. The use of raised nets lowered the chance of ground scavengers feeding on remains. In addition, we inspected the soil and plant litter within a radius of approximately 10 m around each nest tree for prey remains and regurgitated pellets that may have fallen outside the nylon shade cloth.

A Bushnell camera trap (CT) with infrared motion detectors (Models: 119936C, 119537, 119837) was hoisted by ropes to the crown of each nesting tree and positioned approximately 5–10 m away from, and aimed towards, the nest. The CTs were programmed to take three pictures every 10 min or one picture and a 10 s video every 5 min when they were triggered by wind movement or when something moved in front of them. Occasionally, in some months it was not possible to obtain photographic records due to battery failures, equipment malfunctions, or changes in the CT’s positioning due to wind and rain.

The nests were visited by the team monthly e whenever we collected prey remains from soil and nylon shade, we also downloaded data from the CTs and changed their batteries and memory cards.

### Identifying prey in remains

We examined the body parts of the collected prey remains to identify each item to the most specific taxonomic level possible. We identified bones, teeth, claws, and feathers by their external morphology through comparisons to zoological collections, guides, and the literature to identify the species^35–42^. We identified hairs through trichology, which involves analyzing the characteristics of the hair’s cuticle and medulla. We captured close-up images using the Leica LAS EZ 3.0 software and compared them with images from the *Guia de Identificação de Pelos de Mamíferos Brasileiros* (Hair Identification Guide for Brazilian Mammals)^43^ and other literature to identify the species^44,45^. When the morphological analyses only enabled us to identify prey at the genus level, we consulted available species lists for the nests’ region to identify the most likely candidate^46–49^.

We determined the minimum number of individuals per prey species consumed based on the quantity of cranial, mandibular, and pelvic bones and the maximum number of teeth and nails for an individual. In some cases of species without cranial or mandibular bones present, we used the thoracic or pelvic limbs, such as humeri and femora. In these cases, the events were individualized considering the paired bones (right and left). In the case of fragmented crania, mandibles, and pelves, we fit all the parts that appeared to be complementary to the various samples for each nest.

### Identifying prey from CT photographs

The CTs were often triggered by eagles arriving to their nests holding prey in their talons. We used the resultant images to identify prey species by observing the coloration, size, and form of the captured prey. We compared the images to those of the species previously found in the region to identify the prey as precisely as possible, in accordance with Emmons and Feer^50^ and Reis et al.^51^. Records of a prey species in the same nest and within an interval of 24 h were considered a single record. We calculated the records effort based on the number of CTs multiplied by the number of sampling days, where each day corresponded to a 24 h period. Records success was expressed as a percentage determined by the number of records divided by the records effort and multiplied by 100^52^. Because of considerable overlap between photographic and remains data, we did not combine results for the number of individuals per species identified from remains and photographs.

### Prey characterization

We categorized each identified mammal species by: its habits, based on the foraging attributes described by Wilman et al.^53^, such as main diet (folivorous, frugivorous, omnivorous, carnivorous, or insectivorous), predominant foraging stratum (arboreal, scansorial, or terrestrial), activity period (diurnal, nocturnal or cathemeral); mean adult body weight in kilograms (kg), according to Wilman et al.^53^; and to its conservation status at the global^54^, national^12^, and regional levels and in BA^18^ and ES^55^. Based on Eisenberg and Redford^56^, we classified mammals by size: small (< 1 kg), medium (1–10 kg), and large (> 10 kg). The percent contribution of each prey species to the total biomass of the Harpy Eagle diet was calculated as the percentage of potentially consumed biomass (PB = ((N * Pm)/∑ (N * Pm)) * 100), where N represents the number of consumed prey and Pm signifies the average weight of each species.

To compare species richness between the nests in BA and ES and assess whether our sampling efforts were sufficient to represent the assemblage of prey consumed by the Harpy Eagle's in these areas, we used species accumulation curves^57,58^ and the Jackknife-1 estimator^57^ with the EstimateS 9.1 software^59^ for the two methods used in the study. We generated the species accumulation and species richness projection plots using the R programming platform^60^. It is worth noting that ES contains more sampled nests when compared to BA and that, as mentioned previously, the nests had different stages of reproduction.

Based on the list of known mammals found in the studied reserves, we indicated which species could be potential prey for Harpy Eagles. We verified the list by Srbek-Araujo and Kierulff^48^ for VNR and SBR, the list by Falcão et al.^46^ for VS-PNHR, and the list by Sánchez-Lalinde et al.^49^ for SB-PNHR. We considered potential prey to be those belonging to the species or genera recorded in the Harpy Eagle’s diet and within the weight distribution of the prey consumed by the Harpy Eagle in this study (Supplementary Table 1).

### Ethical approval

The material analyzed in this work was collected under the licenses SISBIO N° 31457-2 e N° 73444-1 by ICMBio.

### Consent to participate

All authors consent to their participation in this study.

## Results

We monitored the seven Harpy Eagle nests monthly over a period that varied from 15 to 41 months, totaling 275 visits. During these visits, we collected prey remains below the nests on 152 occasions, while on 123 visits we did not find any prey remains (Table 1). We collected a total of 94 regurgitated pellets below the monitored nests. The cameras recorded 2170 events in total. Among these events, 285 photographic records revealed the presence of prey brought to the seven nests, ranging from 1 to 147 records per nest (Table 1). The variation in the number of events recorded between nests occurred mainly due to battery failures, equipment malfunctions or changes in the CT position due to wind and rain.

### Prey species richness and composition

We identified at least 17 species in the Harpies’ diet with the two methods we used. Sixteen of them are mammals and one is bird that were not identified at the species level, only at the family level (Psittacidae) (Table 2). In both methods, we identified 14 mammal species. Two were identified only in the photographic records (the golden-bellied capuchin (*Sapajus xanthosternos*) and the nine-banded armadillo, (*Dasypus novemcinctus*)), and two others were identified only in the trace analysis (the red-rumped agouti (*Dasyprocta leporina*) and the common tapeti (*Sylvilagus brasiliensis*)) (Table 2). With regard to remains belonging to the Psittacidae family, we recorded two events (Table 2), which may represent one or even two parrot species, although we were unable to identify them at the species level.

**Table 2:** Prey consumed by Harpy Eagle in the seven active nests in the Central Atlantic Forest Ecological Corridor.

Class	Species	Diet	Stratum	Activity	Weight (kg)	Prey remains													
						ES						BA					Total ES + BA	%	PB
Order /Family						VNR-01	VNR-02	VNR-03	SBR-01	Total	PB	VS-PNHR-01	VS-PNHR-02	SB-PNHR-01	Total	PB			
Mammalia																			
Pilosa/Bradypodidae	*Bradypus variegatus*	Fo	Ar	C	4.335	16	1	0	11	28	31.6	46	33	5	84	74.4	112	37.6	55.6
Pilosa/Myrmecophagidae	*Tamandua tetradactyla*	In	Sc	C	5.515	0	0	0	0	0	0	0	0	1	1	1.1	1	0.3	0.6
Primates/Atelidae	*Alouatta guariba* ^VU:1; EN:3; CR:2;4^	Fo	Ar	D	5.188	9	1	1	3	14	18.9	1	4	0	5	5.3	19	6.4	11.2
Primates/Cebidae	*Sapajus robustus* ^EN:1;2;3;4^	Om	Ar	D	2.500	33	0	6	5	44	28.6	4	6	0	10	5.11	54	18.1	15.4
Primates/Cebidae	*Sapajus xanthosternos* ^CR:1; EN:2;4^	Om	Ar	D	2.687	0	0	0	0	0	0	0	0	0	0	0	0	0.0	0
Primates/Pitheciidae	*Callicebus personatus* ^VU:1;2;3^	Fr	Ar	D	1.349	4	0	0	1	5	1.7	0	0	0	0	0	5	1.7	0.7
Primates/Pitheciidae	*Callicebus melanochir* ^VU:1;2;4^	Fr	Ar	D	1.370	0	0	0	0	0	0	1	1	1	3	0.8	3	1.0	0.4
Primates/NI/NI	NI	-	Ar	D	-	0	0	0	0	0	-	0	0	0	0	-	0	0.0	-
Rodentia/Erethizontidae	*Coendou insidiosus*	Fo	Ar	N	1.000	0	0	0	0	0	0	0	0	0	0	0	0	0.0	0
Rodentia/Erethizontidae	*Chaetomys subspinosus* ^VU:1;2;3;4^	Fr	Ar	N	1.299	0	0	0	0	0	0	0	0	0	0	0	0	0.0	0
Rodentia/Erethizontidae	NI^α^	-	Ar	N	-	14	1	3	4	22	-	9	7	6	22	-	44	14.8	-
Rodentia/Dasyproctidae	*Dasyprocta leporina* ^VU:3^	Fr	Te	D	3.020	0	1	0	0	1	0.7	0	0	0	0	0	1	0.3	0.3
Carnivora/Mustelidae	*Eira barbara*	Ca	Te	C	3.910	0	0	0	0	0	0	0	0	1	1	0.7	1	0.3	0.4
Carnivora/Procyonidae	*Nasua nasua*	Fr	Sc	D	3.793	8	1	2	3	14	13.83	1	3	6	10	7.7	24	8.1	10.4
Carnivora/Procyonidae	*Potos flavus*	Fr	Ar	N	3.000	1	0	2	0	3	2.3	0	4	2	6	3.6	9	3.0	3.9
Didelphimorphia/Didelphidae	*Didelphis aurita*	Om	Sc	N	1.163	5	0	1	0	6	1.8	0	1	2	3	0.7	9	3.0	1.1
Lagomorpha/Leporidae	*Sylvilagus brasiliensis*	Fo	Te	N	0.949	1	0	0	0	1	0.2	0	1	0	1	0.1	2	0.7	0.2
Cingulata/Dasypodidae	*Dasypus novemcinctus*	In	Te	N	4.203	0	0	0	0	0	0	0	0	0	0	0	0	0.0	0
Cingulata	NI	In	Te	N	-	1	0	0	0	1	-	0	1	0	1	-	2	0.7	-
NI	NI	-	-	-	-	0	0	0	0	0	-	0	0	2	2	-	2	0.7	-
Aves																			
Psittaciformes/Psittacidae	NI	-	-	-	-	1	0	1	0	2	-	0	0	0	0	-	2	0.7	-
NI	NI	-	-	-	-	4	0	2	0	6	-	1	1	0	2	-	8	2.7	-
Total						97	5	18	27	147		63	62	26	151		298	100	

Class	Species	Diet	Stratum	Activity	Weight (kg)	Camera trap													
						ES						BA					Total ES + BA	%	PB
Order /Family						VNR-01	VNR-02	VNR-03	SBR-01	Total	PB	VS-PNHR-01	VS-PNHR-02	SB-PNHR-01	Total	PB			
Mammalia																			
Pilosa/Bradypodidae	*Bradypus variegatus*	Fo	Ar	C	4.335	15	1	0	12	28	26.7	0	8	0	8	36.9	36	12.5	28.5
Pilosa/Myrmecophagidae	*Tamandua tetradactyla*	In	Sc	C	5.515	2	0	0	0	2	2.4	0	0	1	1	5.8	3	1.0	3
Primates/Atelidae	*Alouatta guariba* ^VU:1; EN:3; CR:2;4^	Fo	Ar	D	5.188	5	0	0	0	5	5.7	0	0	0	0	0	5	1.7	4.7
Primates/Cebidae	*Sapajus robustus* ^EN:1;2;3;4^	Om	Ar	D	2.500	52	7	2	16	77	42.4	1	5	0	6	16	83	28.9	37.9
Primates/Cebidae	*Sapajus xanthosternos* ^CR:1; EN:2;4^	Om	Ar	D	2.687	0	0	0	0	0	0	0	0	1	1	2.8	1	0.3	0.4
Primates/Pitheciidae	*Callicebus personatus* ^VU:1;2;3^	Fr	Ar	D	1.349	6	2	0	2	10	2.9	0	0	0	0	0	10	3.5	2.4
Primates/Pitheciidae	*Callicebus melanochir* ^VU:1;2;4^	Fr	Ar	D	1.370	0	0	0	0	0	0	0	3	1	4	5.8	4	1.0	0.4
Primates/NI/NI	NI	-	Ar	D	-	5	1	0	2	8	-	0	0	0	0	-	8	2.8	-
Rodentia/Erethizontidae	*Coendou insidiosus*	Fo	Ar	N	1.000	9	3	0	2	14	3	0	2	2	4	4.2	18	6.3	3.2
Rodentia/Erethizontidae	*Chaetomys subspinosus* ^VU:1;2;3;4^	Fr	Ar	N	1.299	0	2	0	0	2	0.5	0	1	0	1	1.3	3	1.0	0.7
Rodentia/Erethizontidae	NI^α^	-	Ar	N	-	0	0	0	0	0	-	0	0	0	0	-	0	0.0	-
Rodentia/Dasyproctidae	*Dasyprocta leporina* ^VU:3^	Fr	Te	D	3.020	0	0	0	0	0	0	0	0	0	0	0	0	0.0	0
Carnivora/Mustelidae	*Eira barbara*	Ca	Te	C	3.910	0	0	0	0	0	0	0	0	1	1	4.1	1	0.3	0.7
Carnivora/Procyonidae	*Nasua nasua*	Fr	Sc	D	3.793	8	0	0	7	15	12.5	0	1	3	4	16.1	19	6.6	13.1
Carnivora/Procyonidae	*Potos flavus*	Fr	Ar	N	3.000	0	0	0	0	0	0	0	0	2	2	6.4	2	3.0	0.7
Didelphimorphia/Didelphidae	*Didelphis aurita*	Om	Sc	N	1.163	1	1	0	4	6	1.5	0	0	0	0	0	6	2.1	1.2
Lagomorpha/Leporidae	*Sylvilagus brasiliensis*	Fo	Te	N	0.949	0	0	0	0	0	0	0	0	0	0	0	0	0.0	0
Cingulata/Dasypodidae	*Dasypus novemcinctus*	In	Te	N	4.203	2	0	0	2	1.8	0	0	0	0	0	0	2	0.7	1.5
Cingulata	NI	In	Te	N	-	0	0	0	0	0	-	0	0	0	0	-	0	0.0	-
NI	NI	-	-	-	-	44	9	0	25	78	-	0	4	4	8	-	86	30.0	-
Aves																			0
Psittaciformes/Psittacidae	NI	-	-	-	-	0	0	0	0	0	-	0	0	0	0	-	0	0.0	-
NI	NI	-	-	-	-	0	0	0	0	0	-	0	0	0	0	-	0	0.0	-
Total						149	26	2	70	247		1	24	15	40		287	100	

Nests: VNR-01, VNR-02, VNR-03 and SBR-01 in Espírito Santo (ES); and VS-PNHR-01, VS-PNHR-02 and SB-PNHR-01 in Bahia (BA). It shows the main feeding habit (Diet), predominant foraging stratum (Stratum), activity period (Activity), the percentage of potentially consumed biomass (PB) and the frequency of records of prey found in nests by the method of prey remains and camera trap.

*NI* unidentified, *Fo* folivorous, *Fr* frugivorous, *Om* omnivorous, *Ca* carnivorous, *In* insectivorous, *Ar* arboreal, *Sc* scansorial, *Te* terrestrial, *D* diurnal, *N* nocturnal, *C* cathemeral, ^*α*^ *Coendou* *insidiosus* or *Chaetomys* *subspinosus*. Species conservation status: *VU* Vulnerable, *EN* Endangered and *CR* Critically Endangered at global^54^ (1), Brazil^12^ (2), Espírito Santo^55^ (3) and Bahia^18^ (4). No data (-). Species foraging attribute data by Wilman et al.^53^.

Among the mammals preyed upon by the Harpy Eagles, we recorded at least 13 species in the nests in ES and 14 in BA (Table 2). Ten of the species are commonly recorded in the Harpy Eagle’s diet in both regions (Table 2). The other six were exclusive to the Harpy Eagle’s diet in one of the regions. Specifically, the tayra (*Eira barbara*), the coastal black-handed titi (*Callicebus melanochir*), and *Sapajus xanthosternos* were found only in the diet of the Harpy Eagles in BA. Meanwhile, *Dasyprocta leporina* and the Atlantic titi (*Callicebus personatus*) were found only in ES (Table 2). We identified *Dasypus novemcinctus* only in ES; however, there was one identified trace of the Order Cingulata in BA (Table 2). We found bird remains in three nests in both states (Table 2), but it was not possible to identify them at the species level. In two bird findings inside pellets, one from nest VNR-01 and the other from nest VNR-03 in ES, contour feathers allowed us to identify them down to the family level, Psittacidae (Table 2). We had no photographic record of the Harpy Eagles arriving at the nest with birds as prey.

**Figure 3:**
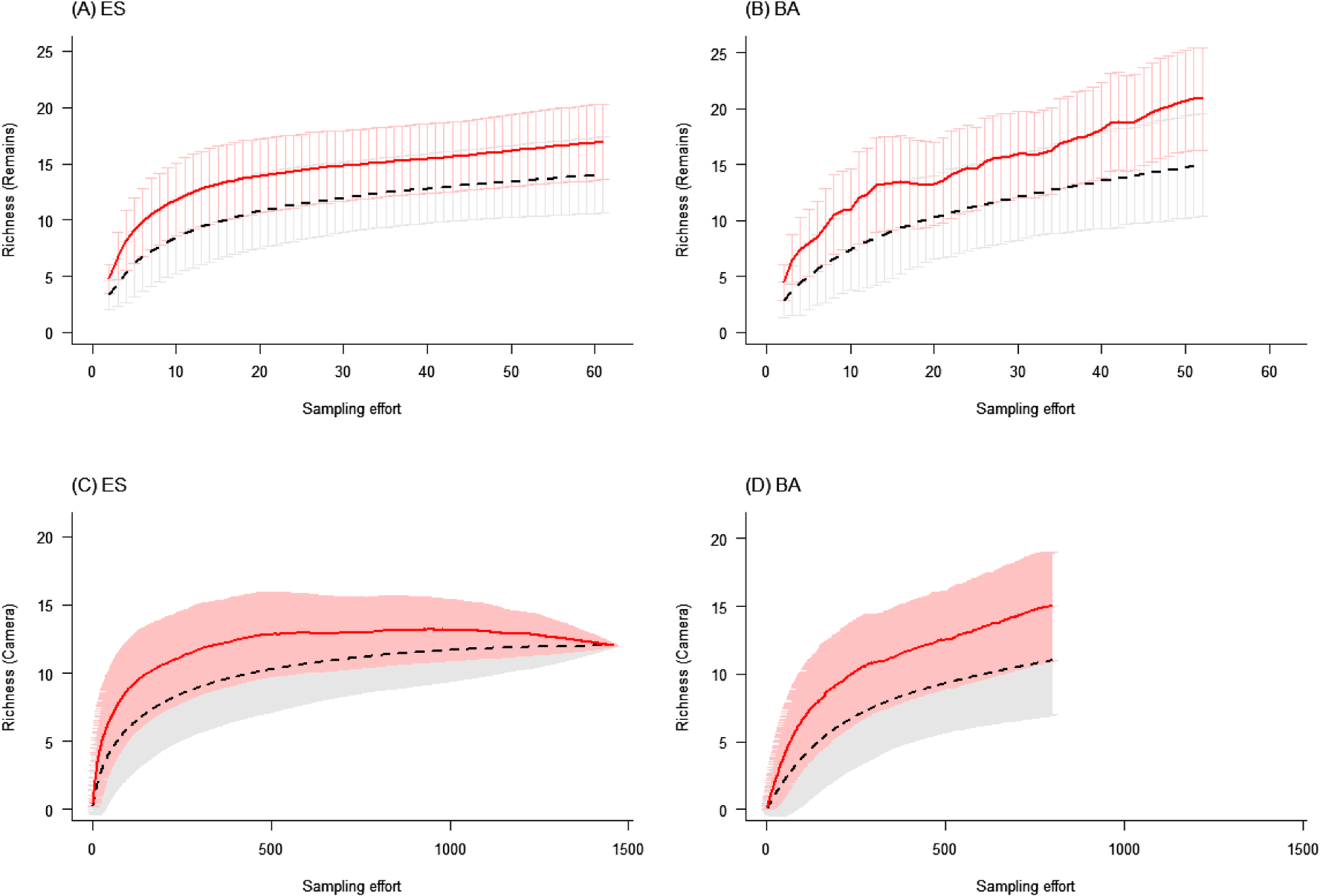
Richness accumulation curve observed (dashed black line) and estimated by Jackknife1 (continuous red line) of the species preyed on in the four Harpy Eagle nests located in the Espírito Santo region (**A** and **C**) and in the three nests in the Bahia region (**B** and **D**). The curves in a and b represent the results found through the methodology of analysis of prey remains found below the nests and in c and d through the camera trap records.

The accumulation curves of prey species did not stabilize in either region (Fig. 3A–D). Using the remains data, Jackknife-1 estimated 17 (± 1.8) species for ES (Fig. 3A) and 21 (± 2.8) for BA (Fig. 3B). When we compared the observed values with the values estimated by Jackknife-1, collection efficiency was 82% for ES and 71% for BA. Using the photographic records, Jackknife-1 estimated a total of 12 (± 0) species for ES (Fig. 3C) and 15 (± 2.1) for BA (Fig. 3D). When we compared the observed values with the values estimated by Jackknife-1, collection efficiency was 100% for ES and 73% for BA.

There are 36 known species of medium-sized and large mammals in VNR and SBR^48^, 33 in VS-PNHR^46^, and 22 in SB-PNHR^49^. In all, the assemblage of medium-sized and large mammals recorded in the study area consists of 45 species (Supplementary Table 1). Out of these species, 25 fall within the range of weights that made up the Harpy Eagle’s diet in this study, 28 are members of the genera or species recorded as prey in other regions, and 22 species fall in both parameters (Supplementary Table 1). In total, there are 31 potential prey species for the Harpy Eagle in the CAFEC, and this study was able to confirm 16 (52%) (Supplementary Table 1).

Among the 16 mammal species consumed by Harpy Eagles, seven (43.7%) are threatened with extinction: five at the global (37.5%), six at the national (37.5%), and seven at the regional (43.7%) levels (Table 2).

### Prey taxa frequency

We identified 298 individuals from the remains analysis and 287 individuals from the photographic records of the nests (Table 2). From the remains, 288 were mammals and 10 were birds, while all the photographic records were of mammals.

We identified the Bahia porcupine (*Coendou insidiosus*) and bristle-spined porcupine (*Chaetomys subspinosus*) from the morphological differences between their spines in the remains and from photographs. However, when we found bone remains of porcupines, it was not possible to differentiate them at the species level. Thus, we quantified the records from the cranial and non-cranial bones of these species as belonging to the Family Erethizontidae, and we did not quantify the number of individuals of the species. The same occurred for the remains belonging to two armadillo individuals. We could not identify them at a more specific taxonomic level, so we only quantified them as belonging to the Order Cingulata (Table 2). However, we were able to quantify the porcupine and armadillo individuals at the species level based on the photographic records (Table 2).

The remains analyses indicated that *Bradypus variegatus* was the most frequent species among the Harpy Eagle’s prey (37.6%), followed by *Sapajus robustus* (18.1%) and species of the Family Erethizontidae (14.8%) (Table 2). The photographic records showed that *Sapajus robustus* was the most frequent prey (23.4%), followed by *Bradypus variegatus* (12.5%) and the South American coati (*Nasua nasua*) (6.6%) (Table 2). Eight taxa identified from the remains and six identified from the photographs presented a relative frequency of ≤ 1% in the Harpy Eagle’s diet (Table 2).

*Bradypus variegatus* was consumed most frequently in BA, constituting 56% of the remains and 20% of the images, while in ES it constituted 19% of the remains and 11.3% of the images (Table 2). In ES, *Sapajus robustus* was the most frequent species found in the remains (30%) and in the images (31.2%), while the frequency of this species in BA was 7% in the remains and 15% in the images (Table 2).

### Attributes of the prey species

**Figure 4:**
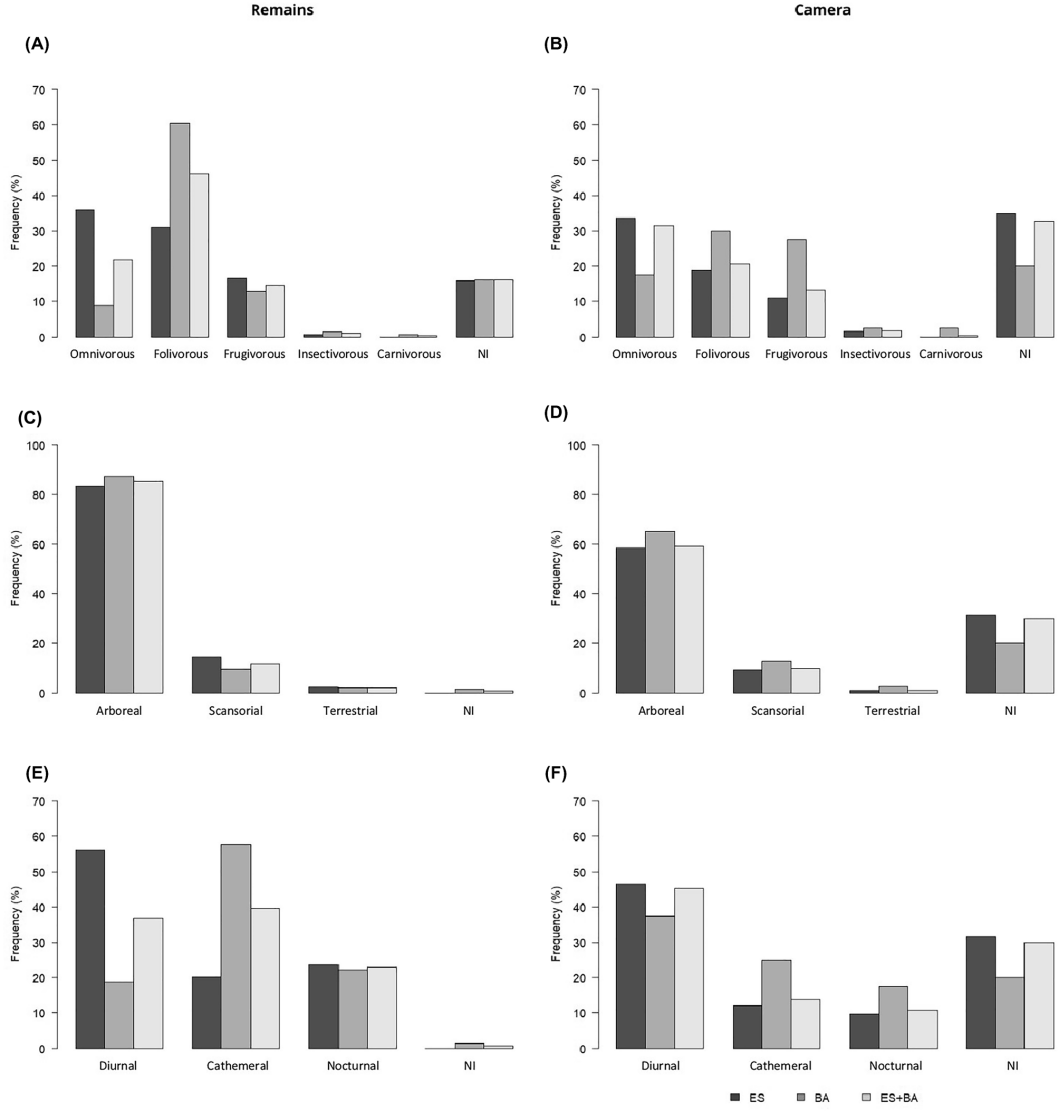
Frequency of Harpy Eagle prey individuals in relation to their diet, foraging stratum and period of activity in both methodologies used in the study, collection of prey remains (**A**, **C** and **E**) and monitoring through cameras trap (**B**, **D** and **F**). Unidentified (NI).

The majority of the species recorded in the remains were medium-sized, arboreal, frugivorous, and nocturnal mammals (Supplementary Fig. 1). However, when taking into account the number of individuals of each species, the majority were medium-sized, arboreal, folivorous, and diurnal or cathemeral mammals (Fig. 4). In the photographic records, the majority of the species were arboreal, frugivorous, and diurnal mammals (Supplementary Fig. 1). However, when taking into account the number of individuals of each species, medium-sized, arboreal, omnivorous, and diurnal mammals predominated (Fig. 4).

**Figure 5:**
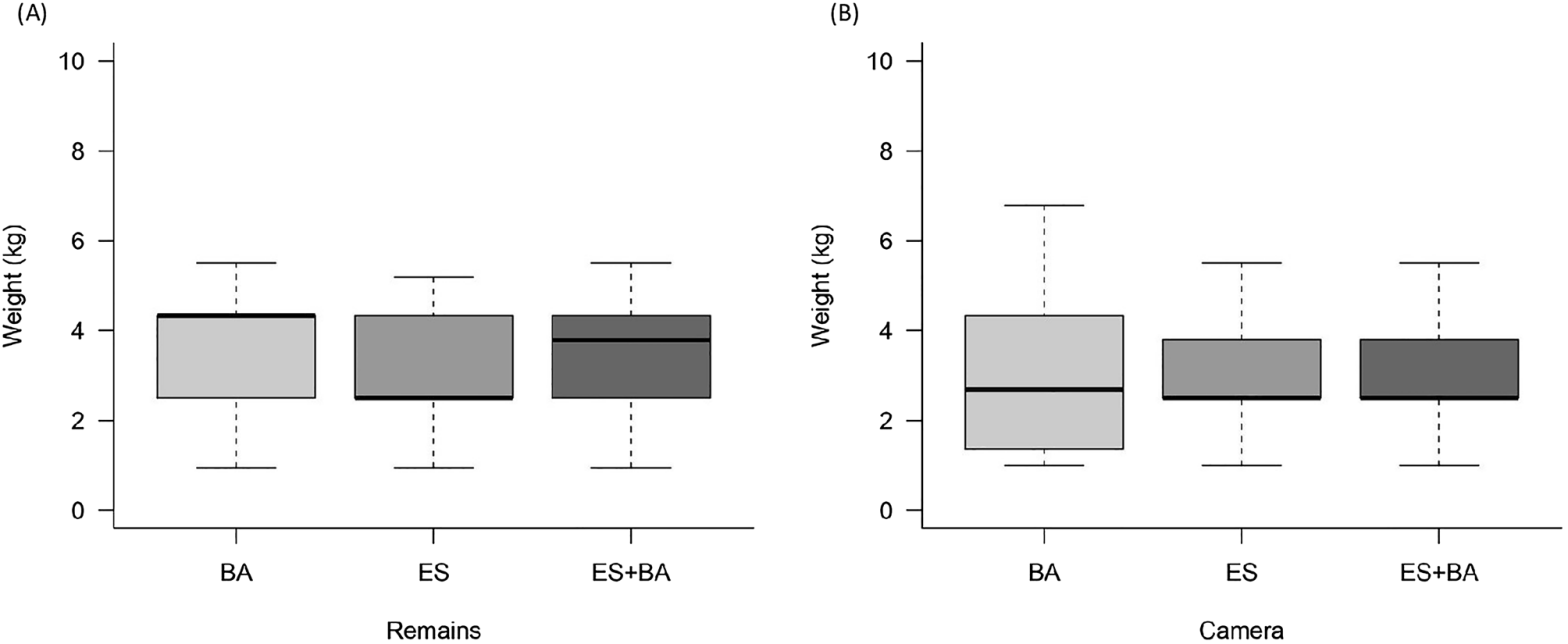
Distribution regarding the weight (kg) of Harpy Eagle prey in relation to the methodologies used in the study and between the regions studied for each method (**A** and **B**).

The mean bodyweight of the prey species we identified ranged from 0.949 to 5.515 kg for *Sylvilagus brasiliensis* and *Tamandua tetradactyla*, respectively (Table 2). Considering the average bodyweight and frequency of each adult prey species, the mean prey bodyweight in the entire study area was 3.637 kg (± 1.1) and 2.834 kg (± 1.2) (Fig. 5), based on remains and camera data, respectively. Equivalent values for ES were 3.309 kg (± 1.2) and 2.815 kg (± 1.1), and for BA were 3.943 kg (± 0.9) and 2.929 kg (± 1.3). Among the 16 mammal species found in the Harpy Eagle’s diet, 15 (93.8%) were categorized as medium-sized (1–10 kg) and 1 (6.2%) (*Sylvilagus brasiliensis*) was small (< 1 kg). *Bradypus variegatus* and *Sapajus robustus* presented the highest percentages of potentially consumed biomass (PB), totaling 55.6 and 15.4% in the trace analysis and 28.5 and 37.9% in the photographic records, respectively.

## Discussion

This study constitutes the first systematic survey of the prey consumed by Harpy Eagles in their only known active nests in the Atlantic Forest. The high habitat quality of protected areas of the CAFEC may explain the presence of Harpy Eagle nests, which requires the availability of area, nesting trees, and prey. The northern ES and southern BA forests are within the phytogeographic region called Tabuleiro Atlantic Forest (TAF)^61^. It was one of the last territories in the Atlantic Forest to be exploited by the current cultural and socioeconomic model, which has generated a great loss in forest cover^62^. Although some nests are close to degraded areas such as pastures, highways and papaya, coffee or cocoa cultivation, the region still hosts a relevant portion of biological richness and has a rich evolutionary history^63^. It contains the largest fragments for mammal conservation in the Atlantic Forest^64^.

### Prey diversity

The protected areas located in BA (VS-PNHR and SB-PNHR) are smaller in size than those in ES (VNR and SBR). Moreover, the reserves of ES altogether form the largest existing contiguous remnant forest fragment of the TAF in the same drainage basin, approximately 50,000 hectares^65^. Given the positive relationship between species richness and area size^66^, we expected the number of prey species for the Harpy Eagle to be higher in ES. Additionally, the number of nests and our efforts to collect remains and photographic records were more considerable in ES than in BA (Table 2). However, our results demonstrated that the species richness of the Harpy Eagle’s diet was similar between both states. This may be explained by the fact that the reserves in BA are surrounded by extensive forest cover that stretch beyond their boundaries, and they are about 197 km apart in two drainage basins. This may also explain the variability in the species richness of the regions we sampled, despite the reserves in BA being smaller than those in ES. Nevertheless, we detected the highest number of prey species in the three VNR nests in ES (13), followed by the two VS-PNHR nests in BA (10).

Most of the species in the Harpy Eagle’s diet in BA and ES were similar. Even though we observed only six different species between both regions, three (*Eira barbara*, *Dasypus novemcinctus*, and *Dasyprocta leporina*) are found in all four protected areas in which the study was conducted^46,48,49^, suggesting that these species rate high as potential prey for Harpy Eagles in all reserves.

Out of the potential prey belonging to species or genera that have previously been recorded in the Harpy Eagle’s diet and are found in the region, *Cabassous tatouay*, *Cabassous unicinctus*, *Dasypus septemcinctus*, *Cerdocyon thous*, *Procyon cancrivorus*, *Leopardus pardalis*, *Leopardus guttulus*, *Leopardus wiedii*, *Tayassu pecari*, *Mazama americana*, *Mazama gouazoubira*, and *Cuniculus paca* were not recorded in this study (Supplementary Table 1). However, it is important to emphasize that Harpy Eagles tend to prey on young individuals of species that reach large body sizes as adults, such as species in the genera *Mazama* and *Tayassu*^67–69^. Furthermore, of the species whose genera have not been confirmed in the Harpy Eagle’s diet but could be considered potential prey due to their size (1–10 kg), we did not observe the following three species: *Euphractus sexcinctus*, *Galictis cuja*, and *Conepatus semistriatus*.

A large proportion of the potential Harpy Eagle prey species were present in protected areas are strictly terrestrial, but only 17.7% were found in the Harpy Eagle’s diet in this study (Supplementary Table 1). The inclusion of terrestrial prey in this predator's diet is often associated with the presence of areas of open vegetation or secondary forests close to the nest, such as pastures, plantations or clearings^70^. In this study, only nests VNR-01, VNR-02, VS-PNRH-02 and SB-PNRH-01 registered exclusively terrestrial prey, which, incidentally, are nests that are located close to pasture and plantation areas. Five armadillo species were considered potential prey: *Cabassous tatouay*, *Cabassous unicinctus*, *Dasypus novemcinctus*, *Dasypus septemcinctus*, and *Euphractus sexcinctus* (Supplementary Table 1). Camera trap recordings showed only *Dasypus novemcinctus* in ES. However, at least two other armadillo species may have been the two individuals found among the remains in the study area, but we were unable to identify the remains at the species level. Therefore, such traces may belong to species of the Family Chlamyphoridae or Dasypodidae.

The only potential arboreal prey we did not find in the remains below the nests was *Sapajus xanthosternos*, but we identified an event in the photographs in which an individual was brought to a nest in the SB-PNHR. Sánchez-Lalinde et al.^26^ had already suggested *Sapajus xanthosternos* as potential prey for the Harpy Eagle in the SB-PNHR. Furthermore, between the years 2013 and 2015, predator–prey interactions between the Harpy Eagle and *Sapajus xanthosternos* were observed in the Una Biological Reserve, which is about 50 km away from the SB-PNHR, but without predatory success^71^.

Sánchez-Lalinde et al.^26^ also suggested six other potential mammalian prey for the Harpy Eagle in the SB-PNHR. We confirmed three in this study: *Nasua nasua*, *Bradypus variegatus*, and *Callicebus melanochir*. Although they are arboreal, we did not consider the other species indicated by Sánchez-Lalinde et al.^26^, Wied’s marmoset (*Callithrix kuhlli*), the golden-headed lion tamarin (*Leontopithecus chrysomelas*), and the painted-tree rat (*Callistomys pictus*), to be potential prey in this study (Supplementary Table 1). They weight less than the species we found (Supplementary Table 1), and their genera, *Callithrix*, *Leontopithecus*, and *Callistomys*, have not been observed in the Harpy Eagle’s prey in previous studies. Moreover, the presence of *Leontopithecus chrysomelas* and *Callistomys pictus* were not confirmed in the list of mammals of the SB-PNHR provided by Sánchez-Lalinde et al.^49^.

In 1991, Galetti et al.^22^ recorded a Harpy Eagle at the Pau-Brasil Experimental Station, an area annexed to the VS-PNHR, and suggested that the white-headed marmoset (*Callithrix geoffroyi*), the tufted capuchin (*Cebus apella* syn. *Sapajus robustus*), and the brown-throated sloth (*Bradypus variegatus*) are potential prey for the Harpy Eagle. When the same authors recorded an individual of *Bradypus variegatus* preyed upon by a Harpy Eagle in the VNR, they indicated that *Cebus apella* (syn. *Sapajus robustus*), *Alouatta guariba*, *Callicebus personatus*, *Callicebus geoffroyi*, and *Potos flavus* are preyed upon by large raptors so are potential prey for the Harpy Eagle. Except for *Callithrix*, which did not fall into our parameters for potential prey, we confirmed all the other genera to be in the Harpy Eagle’s diet in the VS-PNHR and VNR in this study.

In the Atlantic Forest of Argentina, Anfuso et al.^29^ found two species that had not been previously recorded in the Harpy Eagle’s diet: a primate, *Cebus nigritus* (syn. *Sapajus nigritus*), and a feline, *Leopardus wiedii*. Species of the genus *Sapajus* have since been recorded in this and other studies. Although species of the genus *Leopardus* are known to be present in our study area, they were not recorded as prey. Because the Harpy Eagle demonstrates high acceptance for domestic rabbits as prey in captivity, Anfuso et al.^29^ suggested that *Sylvilagus brasiliensis* have high potential as prey in the wild. We confirmed this hypothesis. Though the species presented a low frequency, we recorded two individuals of *Sylvilagus brasiliensis* in the Harpy Eagle’s diet, one in ES and another in BA.

This study has added nine species which had not yet been recorded in other regions to the Harpy Eagle’s known diet^7,9–11,15,33^: five primate species, *Alouatta guariba*, *Sapajus robustus*, *Sapajus xanthosternos*, *Callicebus personatus*, *Callicebus melanochir*; two rodents, *Chaetomys subspinosus* and *Coendou insidiosus*; one marsupial, *Didelphis aurita*; and one lagomorph, *Sylvilagus brasiliensis*. Except for *Sylvilagus,* different species of the same genera had been previously recorded in the Harpy Eagle diet in other regions.

Although a great diversity of reptiles is present in the study area of this investigation^72,73^, no reptile species were recorded. Reptiles were the least common items recorded in the Harpy Eagle’s diet in other studies^9,14,33,67,69,74^. The study area also contains a large diversity of birds^23,72,75^, including species belonging to groups that are more common in the Harpy Eagle’s diet in Central and South America, such as the Galliformes and Piciformes^9–11,14,15,33,69,76^. Nevertheless, we found only one Psittacid and other unidentified remains. Even so, the frequency of birds in the Harpy Eagle’s diet in this study (3.3%) was like that of other studies conducted in different regions^6,7,33^.

### Attributes of the prey species

In this study, mammal species, particularly arboreal mammals, made up the majority of the Harpy Eagle’s diet (Table 2). Studies conducted in other regions found similar results^7,10,11,15,33^. For example, Muñiz-López et al.^15^ monitored 11 Harpy Eagles nests in Ecuador and found that 84% of the mammal prey consumed by Harpy Eagles were arboreal species. In Parintins, Amazonas, Aguiar-Silva et al.^11^ found that arboreal mammal prey made up 99% of the Harpy Eagle’s diet in five nests.

The Harpy Eagles also preyed on species that forage exclusively in the terrestrial stratum, but these species occurred less frequently in the diet. The following species were classified as terrestrial: *Dasyprocta leporina*, *Dasypus* sp., *Sylvilagus brasiliensis*, and *Eira barbara*^53^. However, other authors do not consider *Eira barbara* to be an exclusively terrestrial species, as the species was seen in or near the canopy layer in one in four direct observations^77^. Moreover, there has been one record of the species visiting the interior of a Harpy Eagle’s nest in the Cerrado^78^. According to Aguiar-Silva et al.^33^, records ground-dwelling species such as armadillos in the Harpy Eagle’s diet may indicate that the Harpy Eagle also forages in open areas, possibly at the edge or in the matrix of forest fragments, and in the low vegetation and clearings of reserves. Nonetheless, it would likely be difficult for only terrestrial prey to maintain Harpy Eagles in the wild, considering their preference and specialization for arboreal prey.

There is a relationship between the foraging stratum and diet of the prey species. Folivorous, frugivorous, and omnivorous prey species were primarily arboreal, while insectivorous or carnivorous species were primarily terrestrial. Thus, it is likely that the low capture frequency of insectivorous and carnivorous prey is due to their foraging stratum, since obscurement by the understory likely interferes with the Harpy Eagle’s ability to find and hunt prey^67^. Consequently, herbivorous arboreal animals are visually and acoustically more accessible to the predator^67^.

The Harpy Eagles in our study preyed on cathemeral species. Since the Harpy Eagle’s activity period is diurnal, it is likely that nocturnal prey is captured when the individuals move during periods when they exhibit greater lethargy^10^ or sleeping, thus facilitating capture. Miranda et al.^79^ associated the rise in the predation rates of nocturnal mammals with darker nights, when nocturnal prey species such as anteaters, opossums, and armadillos are more active. Our camera-monitoring efforts in the seven nests did not record events in which the Harpy Eagle arrived at the nest with prey during the twilight or nighttime period. As for predation on diurnal species, the Harpy Eagle possibly takes advantage of moments when the prey is more active, such as when foraging, as this is when they are most visually and acoustically detectable^67^. At the same time, the Harpy Eagle has a more precise and enhanced predation strategy in comparison to other raptors. They possess a combination of extremely acute vision and a retractable facial disc which favors visual and acoustic detection^79^. These features make it capable of locating even relatively cryptic prey.

The species that contributed the most biomass to the Harpy Eagle’s diet in the region studied were *Bradypus variegatus* and *Sapajus robustus*; both species are medium-sized (1–10 kg). While this study adopted the body weight of adult mammalian prey species provided by Wilman et al.^53^, the mean weight of consumed prey was similar to that estimated by other authors who applied other methods to estimate prey weight. The mean prey weight (2.834–3.637 kg) in this study was similar to that of studies conducted in the Amazon Rainforest, which ranged from 2.6 kg^11^  to 4 kg^4^. Harpy Eagles do not capture only adult prey, juvenile and sub-adult individuals are also part of the Harpy Eagle’s diet^11,67,80^. Therefore, since we used adult weights, our results may be an overestimation, but we believe they still adequately demonstrate the size of the Harpy Eagle’s prey.

The Harpy Eagle’s diet is apparently opportunistic and adaptive; it allows them to sustain themselves in impacted forests by feeding on disturbance-tolerant prey^76,80^. Raptors species with less specialized diets are able to survive and forage in more degraded areas, such as cabrucas^81^. Because the conservation status of the Atlantic Forest is worse than that of the Amazon, the frequency of arboreal prey consumption in the Atlantic Forest may be lower than in the Amazon. Furthermore, forest loss is directly linked to severe reductions in prey capture rate and biomass^14^. However, the results found herein indicate that the Harpy Eagle in protected areas of the CAFEC still maintains its diet within the expectations of its specialized predation, even with nests very close (< 1 km) to pasture and crop areas, such as the VNR-01 and SB-PNHR-01. The fauna and flora of the TAF in the CAFEC is often compared to the Amazon Rainforest^61,82^. However, these characteristics are not present throughout the extent of the Atlantic Forest. The region’s environmental conditions and conservation status vary greatly throughout its distribution, as does species composition^83^. Therefore, it is not possible to extrapolate the results found herein to the Harpy Eagle’s entire distribution in the Atlantic Forest. Although there have been recent records of Harpy Eagles in the southern Brazilian states of Rio Grande do Sul^28^ or even in the mountainous region of Espírito Santo^25^ in the CAFEC, no other nests have been found in the Brazilian Atlantic Forest recently.

### Abundance and conservation status of the prey species

Except for *Bradypus variegatus* and *Coendou insidiosus*, all of the major arboreal mammal species that make up the Harpy Eagle’s diet in this study are threatened with extinction. This is a troubling factor since Harpy Eagles depend on arboreal mammals for food. The scarcity of these prey can interrupt natural food chains and force the predator to look for food alternatives, which can lead to food overlap with other species with which it did not previously compete for the same food resources^32^. *Bradypus variegatus* was the species that contributed the most in terms of biomass to the Harpy Eagle’s diet in our study. It is also one of the species that Harpy Eagles consume the most in other regions^10,11,15,33,67,69^.

This can be explained by the prey’s own behavior. The species is commonly found in the upper canopy trees of the forest eating and exposing itself to the sun, and it can stay between one and three days in the same tree^37,68,80^. The species’ bodily movements are slower than those of the other prey species identified in the study, such as primates, so it is easier to capture. However, the Harpy Eagles preyed more upon *Bradypus variegatus* in BA but preyed more upon *Sapajus robustus* in ES. Such differences may indicate that these prey species’ abundance between the regions in ES and BA may be different, with a greater abundance of *Bradypus variegatus* in BA and of *Sapajus robustus* in ES. It is worth noting that the SB-PNHR-01 nest, the only one with the presence of eaglet in BA during the study, recorded porcupine and coatis prey more frequently. This difference can be explained by the area where the nest is located, close to the cabrucas.

*Sapajus robustus* is classified as Endangered throughout its distribution. In the VNR, there are an estimated 1725 individuals or a density of 8.025 ind/km^2^^84^. In 2021, the Federal Police arrested a gang that killed mothers and captured the babies of *Sapajus robustus* in the VNR and SBR for trafficking. In BA, studies involving the loss of area potentially occupied by *Sapajus robustus* due to forest fragmentation showed a 50.7% decrease in suitable habitats for the species between 1995 and 2010^85^. This decrease in the species’ home range may suggest a strong decline in population density in BA, and it may explain its lower occurrence in the Harpy Eagle’s diet in this region compared to ES. These primates are diurnal and travel in troops, and their howling at dusk, dawn, and when they are moving through the canopy in search of fruit^67^. These features likely make them easy for Harpy Eagles to locate and hunt on a regular basis. Additionally, Harpy Eagles have a habit of capturing younger individuals that are inexperienced at escaping, thus easier to catch^11,78,86^.

*Sapajus xanthosternos* is classified as Critically Endangered globally^87^ and as Endangered in Brazil^12^, and was among the 25 most endangered primates in the world^88^. Its total remaining population size is estimated to be 3000 to 5000 individuals throughout its distribution^89^. A study carried out in the Una Biological Reserve, located in southern Bahia close to the SB-PNHR, estimated a group of 25 individuals occupying a territory of 1030 ha, generating a density of 0.024 ind/ha^90^. The low density found for this species is a result of hunting practices and the pet trade^91–93^. The small size of its population may explain why only record of the species was found in the SB-PNHR.

*Alouatta guariba* is classified as Critically Endangered in Brazil^12^ and BA^18^, Endangered in ES^55^ and Vulnerable by the IUCN^54^. The species’ population is declining, and the current population size is suspected to be extremely small^94^. *Alouatta guariba* represents the genus of primates that lost the most individuals and populations during the yellow fever epidemic in Brazil in 2016, but the outbreak did not reach the protected areas of this study^95^.

*Callicebus personatus* and *Callicebus melanochir,* which were identified as prey in this study, are classified as Vulnerable at all scales of their distribution^12,18,55,96,97^. Throughout the distribution of these two species, estimates indicate that the number of mature individuals is less than 10,000 for *Callicebus personatus*^98^ and probably not much greater than 10,000 for *Callicebus melanochir*^99^. In the VNR, the population size for *Callicebus personatus* is estimated to be 2252 (1768–3252) individuals^100^. In a study on *Callicebus melanochir* conducted in a remnant forest in southern BA, Costa-Araújo et al.^101^ found that the species is more likely to be present in larger, higher-quality forest fragments. The low proportion of these species in Harpy Eagle diets probably reflects the low abundance of these species in the reserves, especially in BA, where the forest fragments are smaller. Moreover, these species are among the lightest arboreal prey (1.359 kg) found in this study.

Out of the two porcupine species consumed by Harpy Eagles, *Chaetomys subspinosus* is rare in the wild^102^, and its population is declining^103^. Furthermore, it is classified as Vulnerable throughout its distribution^12,18,55,103^. *Coendou insidiosus*, on the other hand, is considered Least Concern. The camera-trap data evidenced a total of 18 individuals of *Coendou insidiosus* and three individuals of *Chaetomys subspinosus* across all nests. Therefore, this result likely reflects the low abundance of *Chaetomys subspinosus* relative to *Coendou insidiosus*.

The terrestrial* Sylvilagus brasiliensis* is also threatened, classified as globally Endangered^104^. However, the species is not classified as threatened in Brazil and in the regions where the study was conducted^12,18,55^. Finally, *Dasyprocta leporina* is classified as Vulnerable only in ES, where its main threats are hunting and capture^55^. Both species presented a low frequency in the Harpy Eagle’s diet in the CAFEC, which is probably attributed to the Harpy Eagle's low foraging in the terrestrial stratum and its most frequent consumption of arboreal prey.

In the CAFEC, 50% of the species in the Harpy Eagle’s diet are threatened with extinction, and between 47 and 50% of the potentially consumed biomass are individuals of threatened species (Table 2). Endangered species are experiencing population declines, mainly as a result of habitat loss and fragmentation generated by anthropogenic activities such as livestock farming, agriculture, silviculture, roads, and overexploitation, among others^105^. The Atlantic Forest hosts many mammals that are on the brink of extinction^106^, which includes the identified prey of the Harpy Eagle. The population decline of prey in the CAFEC signifies declining resources for the Harpy Eagle. In addition to threats to Harpy Eagles due to declining nesting sites^79^, poor habitat quality in their home range^14^, and the hunting of individuals^34,74,107,108^, Harpy Eagles may be under even greater pressure as their prey is also under threat^15^.

The limited food resources of its specialization may put pressure on this predator to change the quality of its diet. However, this did not occur in the study area of this investigation. Although many of the Harpy Eagle’s prey are under threat, the Harpy Eagle also preys on species that are not threatened, such as *Bradypus variegatus* and of *Coendou insidiosus*, which may reduce its predatory impact on the populations of prey threatened with extinction^67^. Still, what likely limits the Harpy Eagle from preying on species that are within its specialization and threatened with extinction is their availability in the environment. Additionally, the pressure on threatened species by the Harpy Eagle may contribute to the maintenance of their dangerously low population sizes. We suggest that more studies look into this issue and evaluate interactions between threatened prey and predators and the supportive capacity of reserves in sustaining these ecological relationships in the long term. Such results would be useful in informing conservation strategies for all species involved.

## Conclusion

The presence of Harpy Eagle nests in the CAFEC reserves and the diet presented herein attests to the quality of these forests in maintaining the only currently known Harpy Eagle breeding sites in the Brazilian Atlantic Forest and contribute to knowledge about the Harpy Eagle’s diet. Most species captured by the Harpy Eagle in the region were medium-sized, folivorous, frugivorous, and omnivorous mammals that forage in the upper canopy, a pattern similar to that recorded in the diet of Harpy Eagles in the Amazon. New species have been added to the knowledge on the Harpy Eagle’s diet. However, important species in the Harpy Eagle’s diet are threatened with extinction, making them limited resources in the Atlantic Forest, although some of them were found to be frequent in this predator’s diet. Nevertheless, Harpy Eagles may face an even greater threat due to the population reduction of their prey. The Harpy Eagle is a top predator of the ecological chain and requires large areas with availability and quality of prey for its existence in the medium and long term. Thus, actions focused on its conservation in the reserves for the protection of its nests in the Atlantic Forest, may benefit a range of other threatened species that interact with the Harpy Eagle in the predator–prey relationship.

### Supplementary Information

Supplementary Figure 1.

Supplementary Table 1.

## Data Availability

All data generated or analysed during this study are included in this published article and its supplementary information files.
