# Supplementary material for: The prey of the Harpy Eagle in its last reproductive refuges in the Atlantic Forest
Source: Sci Rep. 2023 Oct 25;13:18308. doi: 10.1038/s41598-023-44014-9 (PMC10600338; doi:10.1038/s41598-023-44014-9)
Supplement: Supplementary file 1 — Supplementary Figure 1. [file 41598_2023_44014_MOESM1_ESM.pdf]

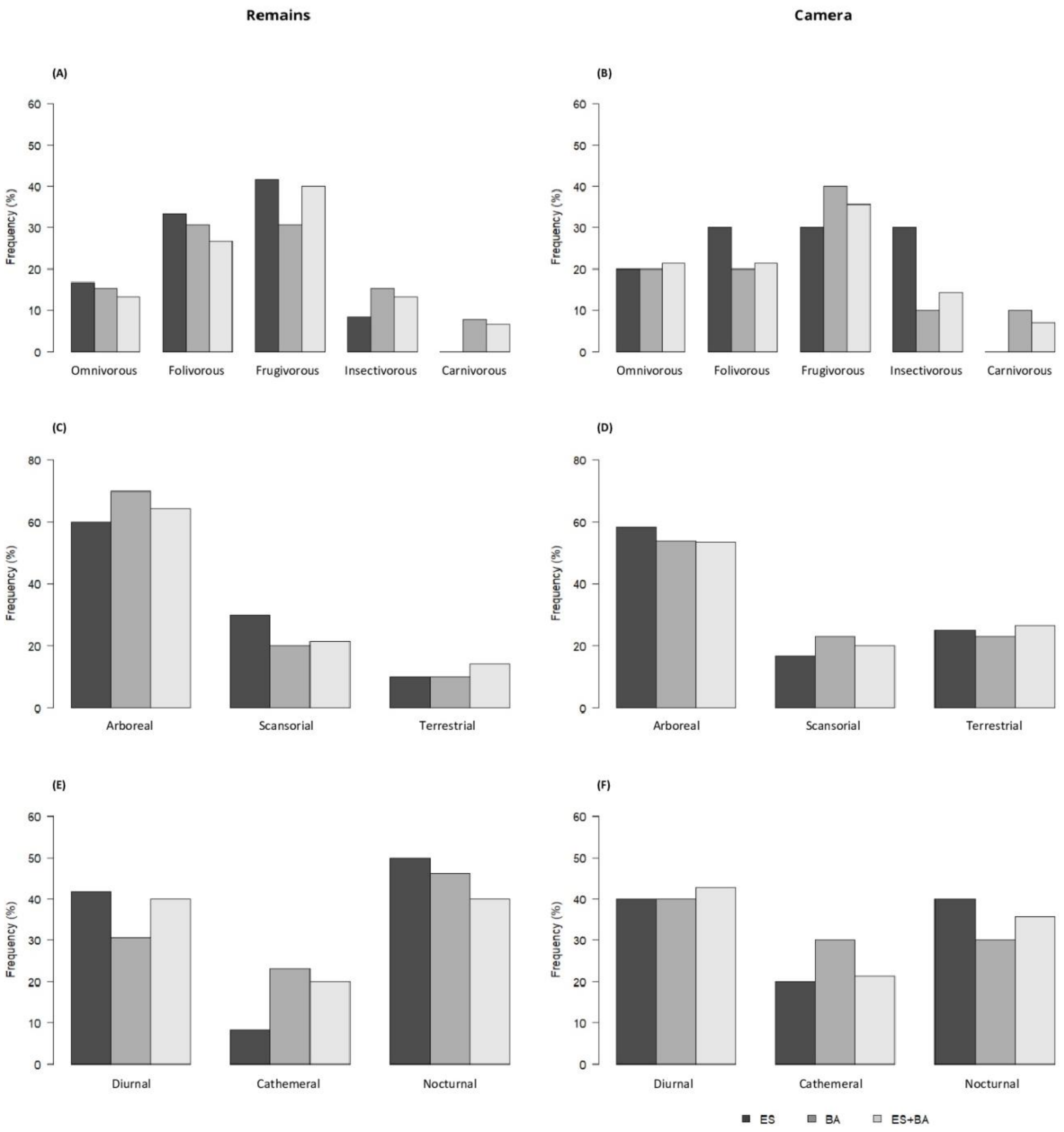

Supplementary Figure 1. Frequency of Harpy Eagle prey species in relation to their diet, foraging stratum and period of activity in both methodologies used in the study, collection of prey remains (A, C and E) and monitoring through cameras trap (B, D and F).
